# Aconitine in Synergistic, Additive and Antagonistic Approaches

**DOI:** 10.3390/toxins16110460

**Published:** 2024-10-27

**Authors:** Nicoleta Anca Şuţan, Alina Paunescu, Carmen Topala, Codruţa Dobrescu, Maria Cristina Ponepal, Diana Ionela Popescu (Stegarus), Liliana Cristina Soare, Radu Tamaian

**Affiliations:** 1 Department of Natural Sciences, National University of Science and Technology POLITEHNICA Bucharest, Pitesti University Centre, 110040 Pitesti, Romania; nicoleta_anca.sutan@upb.ro (N.A.Ş.); alina.paunescu@upb.ro (A.P.); codruta.dobrescu@upb.ro (C.D.); maria.ponepal@upb.ro (M.C.P.); liliana.soare@upb.ro (L.C.S.); 2 Department of Environmental Engineering and Applied Sciences, National University of Science and Technology POLITEHNICA Bucharest, Pitesti University Centre, 110040 Pitesti, Romania; carmen.topala@upb.ro; 3 National Research and Development Institute for Cryogenic and Isotopic Technologies—ICSI Rm. Valcea, 240050 Ramnicu Valcea, Romania; radu.tamaian@icsi.ro

**Keywords:** aconitine, synergistic action, antagonistic effect, combination therapy

## Abstract

Aconitine is a highly poisonous C_19_-diterpenoid alkaloid identified and isolated from the species of the genus *Aconitum.* Aconitine is indicated in the treatment of cardiovascular diseases (CVDs) and, due to its neurotoxic effects, is a very effective drug in pain release. A total of 101 relevant scientific papers were manually searched on the Web of Science, Scopus, Science Direct, Google Scholar, PubMed and Dovepress databases and in the books available in the library of the Department of Natural Sciences, the National University of Science and Technology POLITEHNICA Bucharest, Pitesti University Centre, Romania. In combination treatments, aconitine shows antiarrhythmic and anti-inflammatory activity, a synergistic antiproliferative effect and decreased reactive oxygen species (ROS) generation, an improved biodistribution and bioavailability. Additionally, the entrapment of aconitine in engineered nanoparticles represents a promising method for reducing the toxicity of this alkaloid. This review provides, for the first time, a comprehensive picture of the knowledge and research on the synergistic, additive and antagonistic effects of aconitine in combination treatments applied in vivo or in vitro. The summarized studies represent important clues in addressing the multitude of knowledge, which can find their utility in practical and clinical applications.

## 1. Introduction

Herbal medicine invariably had an important role in people’s lives and act in harmony with their particular environment, either to meet the needs of low-cost primary care [1,2] or to compensate for the lack of access to allopathic medicine treatments, sometimes to alleviate concerns about side effects and to address a more natural form of treatment or as a result of compatibility with their personal and religious beliefs [3]. However, the potential benefits as well as the potential side effects and risks must be considered to obtain safe and effective use profiles.

**Figure 1:**
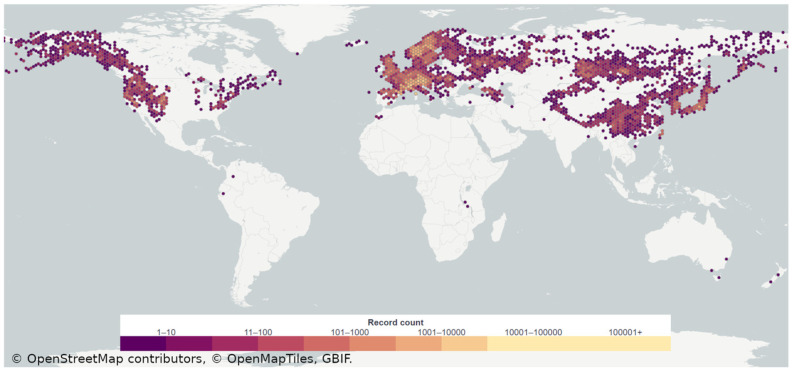
Global distribution of genus *Aconitum*. Plate Carrée map projection using georeferenced records of GBIF—the Global Biodiversity Information Facility (https://www.gbif.org/species/3033663, accessed on 2 February 2024)—map by OpenStreetMap® (Cambridge, UK) contributors (open data, licensed under the Open Data Commons Open Database License by the OpenStreetMap Foundation—the Creative Commons Attribution-ShareAlike 2.0 license: CC BY-SA 2.0), and open-source maps made for self-hosting by OpenMapTiles (open-source license: BSD 3-Clause License and CC-BY 4.0).

“Lurida terribiles miscent aconita novercae” (translation in English: “terrible stepmothers mix up ghastly pale wolf’s bane”), the terrible warning of Publius Ovidius Naso penned in *Metamorphoses* 1.147, has echoed through time since the reign of Augustus (born Gaius Octavius), the first Roman Emperor. Those ominous words foreshadow the enigmatic nature of *Aconitum*, a genus of flowering plants commonly known as aconites, monkshoods, the devil’s helmet, or the wolf’s bane. The genus *Aconitum* unfolds as a botanical marvel, encompassing 471 documented species. *Aconitum* species are commonly occurring in the Northern Hemisphere and can also be found in some isolated niches across South America, South Africa, and Oceania (Figure 1) [4,5].

Aconitine is one of the main secondary metabolites of *Aconitum* sp. and is well known in Chinese traditional medicine, but not exclusively, for being used for the preparation of herbal remedies to prevent or cure various human diseases or metabolic disorders involved in the pathogenesis of these conditions [6]. In traditional medicinal practices, the lateral roots/rhizomes of *Aconitum* sp., which are processed to a significantly reduced toxicity, are most commonly used for their analgesic properties and specific effects on the heart system, heart rate and blood pressure [7,8,9,10,11,12,13]. In alternative homeopathic treatments, a number of formulations containing aconites, such as Qili-Qiangxin capsules, used in Shenfu injections are indicated in the treatment of heart failure [14,15]. The analgesic properties in trigeminal neuralgia and in rheumatic disorders, anticongestive and sedative effects in respiratory diseases, and the action on the motor centers of the young lateral tubers (Aconiti tubera) of *Aconitum tauricum* Wulfen and *Aconitum callibotryon* Reichenb. are also known in Romanian medicine. Dumitru and Dumitru [16] stated that the product must be used only as prescribed by a qualified health professional and indicated the use of 0.01 g of powder once and 0.05 g in 24 h. The decoction of leaves boiled in wine is popularly used to combat coughs and neuralgia, and the grounded roots are mixed with lard to treat infected wounds. Aconite tincture is also part of some medicines, such as Tusomag and Sirogal.

Combination treatments/therapies have encountered growing interest over monotherapy and are consistently used in the medical field due to their increased effectiveness in multifactorial and/or complex disease treatment, in preventing or against multi-drug resistant pathogens [17,18,19], in cancer treatment [20]. Despite the fact that both synergistic and additive effects can be acquired from a multitude of drug combinations, antagonistic effects are more frequently obtained [21]. In phytomedicine, complex mixtures derived from plants are used for the treatment of various ailments [22,23,24,25]. These derivatives of complex combinations can have multi-target unexpected interactions, reducing benefits and increasing risks for patients [26,27].

Aconitine is one of the most studied alkaloids, especially regarding deciphering its mechanisms of action as well as the ways to mitigate its toxicity, for the purpose of expanding its subsequent effective therapeutic uses. Along these lines, we conducted a review of the recent advances in the antagonistic, synergistic and additive effects of aconitine, together with emerging strategies that can offer the possibility of customizing the treatment to obtain the maximum expected effect.

In this review, we provide an overview of the toxic signs of aconitine in in vitro, in vivo and in silico approaches, alone or in combination therapies, and the current trends from plants to nanoparticles. This review highlights the challenges and opportunities for the prevention or mitigation of toxicity, as well as the development of combination therapies with predictable activity. Due to the current small pool of data, this paper would be of interest for cardiologists and clinicians and for other cellular and molecular biology, biochemistry, biomedical research, physiopathology and forensics studies.

## 2. Aconitine

**Figure 2:**
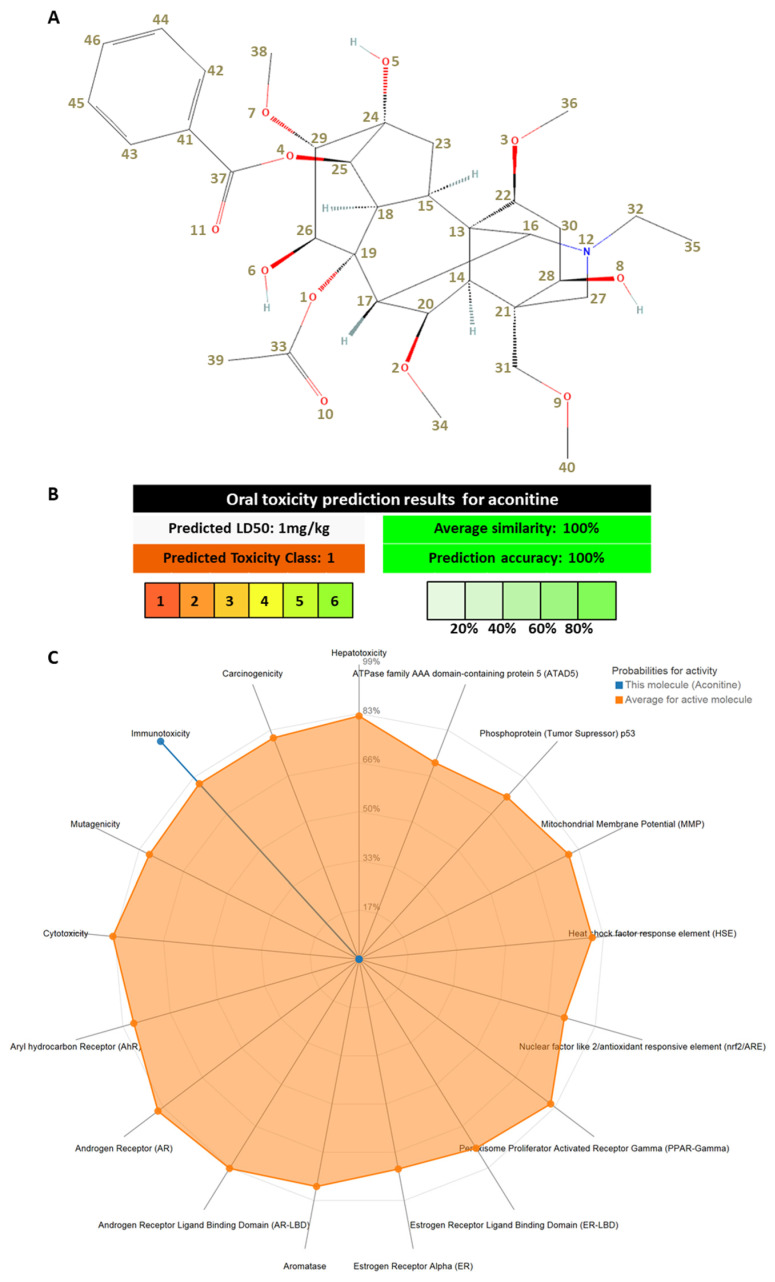
Graphical depiction of aconitine. (**A**) Two-dimensional (2D) structure of aconitine (numbering of atoms was carried out with the freeware version of ACD/Labs ChemSketch 2023.1.2), based on the ideal model provided by the PubChem database [45,46] (for the PubChem CID 245005. (**B**) Predicted LD50 and toxicity class of aconitine performed with ProTox-II webserver. (**C**) Toxicity radar chart of aconitine performed with ProTox-II webserver (illustrates the confidence of positive toxicity results compared to the average of its class).

*Aconitum* genus, belonging to the Ranunculaceae family, comprises numerous herbal medicinal species, which accumulate highly toxic diterpene alkaloids (DAs) [28,29]. These secondary metabolites have attracted considerable attention because of their enthralling chemistry and noteworthy physiological effects. The aconitine, with a complex chemical structure (Figure 2A), is one of the predominant C_19_-diterpenoid alkaloids (C_19_-DAs) identified in various *Aconitum* species (Table 1). While different extraction methods can significantly affect the efficiency of aconitine extraction, the inherent variation in aconitine content is predominantly determined by the species of *Aconitum* and the plant part being extracted for medicinal purposes. Likewise, Yang et al. [30] found that endophytic fungus XJ-AC03 isolated from healthy roots of *Aconitum leucostomum* Worosch. synthesized aconitine when grown in potato dextrose agar medium.

Furthermore, the data presented in Table 1 highlight the specific impacts of extraction methods on the extraction rates of aconitine. For instance, various extraction techniques yield different aconitine concentrations across *Aconitum* species. For example, the extraction of aconitine from *Aconitum szechenyianum* using refluxed acidic alcohol results in the content ranging from 331.7 to 1700 μg/g, indicating high extraction efficiency. Similarly, when extracted, *Aconitum pendulum* yields a content of 296.3 μg/g upon employing a similar extraction method. In contrast, *Aconitum chasmanthum*, extracted via sonication with HCl 0.05 M, shows an aconitine content of 7800–8100 μg/g. These findings accurately reflect how the complex interplay of factors such as *Aconitum* species and plant parts affect extraction rates of aconitine.

**Table 1:** The content of aconitine in various Aconitum species.

Species	Extraction Method/Solvent	Plant Parts	Content	References
*Aconitum carmichaelli* Debx.	Ultrasonic bath/MeOH–H_2_O/MeOH-CHCH_3_/diethyl ether + NH_3_/1% aqueous HCl	Root	50–73 μg/g	Csupor et al. [31]
*Aconitum kusnezoffii* Reichb.	Refluxed/ acidic alcohol solution	Root	69.65 μg/g	Kang et al. [32]
*Aconitum taipeicum* Hand-Mazz		Root	78.47 μg/g	
*Aconitum pendulum* Busch		Root	296.3 μg/g	
*Aconitum szechenyianum* Gay		Root	331.7–1700 μg/g	
*Aconitum chasmanthum* Stapf ex Holmes	Sonication/HCl 0.05 M	Rhizome	7800–8100 μg/g	Jabeen et al. [33]
*Aconitum heterophyllum* Wall. ex. Royle		Rhizome	700–800 μg/g	
*Aconitum japonicum*	HCl 1 mmol/L	Leaf	5.9 μg/g	Kasahara et al. [34]
		Root	928.1 μg/g	
		Petal	46.1 μg/g	
		Nectaries	69.8 μg/g	
*Aconitum violaceum* Jacq. ex Stapf.	Ammoniacal ether, methanol	Stem	81–688 μg/g	Rawat et al. [35]
		Leaf	631–2091 μg/g	
		Root/Tuber	1810–9913 μg/g	
		Bud	10–89 μg/g	
*Aconitum toxicum* Reichenb.	96% ethanol, 96% methanol	Rhizome	4.891–18.211 μg/g	Sutan et al. [24]
		Leaf	7.5 μg/g	Sutan et al. [26]
*Aconitum napellus* ssp. lusitanicum Rouy.	Extraction with tissue homogenizer/70% aqueous methanol and 0.5% formic acid	Pollen	232 μg/g	Jacquemart et al. [36]
		Nectar	0.005 μg/g	

Note: The terms “root” and “rhizome” are often used interchangeably, but they refer to different structures in the plant world. A root is a vital part of a plant that typically grows downward in the soil, anchoring the plant and absorbing water and nutrients. In contrast, a rhizome is a type of underground stem that grows horizontally. It can produce roots and shoots at nodes along its length, allowing plants to propagate vegetatively.

### 2.1. Natural Sources, Chemistry and Toxic Properties of Acontine

Aconitine is known as one of the most toxic and highly effective diester DAs. Intoxication with aconitine can cause numbness, vomiting, nausea, dizziness, palpitation, several ventricular arrhythmias such as ventricular tachycardia, ventricular extrasystoles, ventricular fibrillation, and torsades des pointes [37,38,39,40]. Approximately 1 mg of aconitine could be fatal [41] (Figure 2B).

This toxin is characterized by an acetyl group at C8 and benzoyl ester group at C14, which are responsible for its cardiotoxic and neurotoxic properties [10,11] (Figure 2A,C). Analyzing the cardiac activity–structure relationship of C19-DAs, some of them isolated from *Aconitum* sp. [9] showed that an α-hydroxyl group at C-15, a hydroxyl group at C-8, an α-methoxyl or hydroxyl group at C-1, and a secondary amine or N-methyl group in ring A are important structural features necessary for the cardiac activities of aconitine-type C19-DAs without any ester groups. Previously, it has been shown that the toxicity of aconitine is due to the aroyl/aroyloxy group at the R14 position and the analgesic activity is secondary to its toxic effect [42].

Within this context, the ProTox-II web-server [43] was used for a fast toxicological profiling of aconitine. Utilizing a comprehensive approach, ProTox-II integrates a diverse set of trained models, including molecular similarity, pharmacophores, fragment propensities, and machine learning models. These models were constructed based on data derived from both in vitro assays and in vivo study cases, covering critical toxicity endpoints such as acute toxicity, organ toxicity, and adverse outcome pathways—Tox21 [44]. Specifically, the trained models were designed for acute toxicity, expressed as the lethal dose for oral toxicity (LD50), in alignment with the globally harmonized system of the classification and labeling of hazardous chemicals. Additionally, ProTox-II addresses hepatotoxicity, along with three distinct toxicology endpoints (carcinogenicity, immunotoxicity, and mutagenicity). Furthermore, the models extend to nuclear receptor signaling pathways and stress response pathways, enabling an in-depth evaluation of Tox21 (Figure 2B,C).

Examining Figure 2B, it becomes clear that the 100% average similarity underscores a precise match or exceedingly close resemblance (pertaining to the two-dimensional structure employed as query input in the ProTox-II web server) of aconitine to compounds utilized in the model’s training set. Succinctly, the structure of aconitine bears striking similarity to those compounds acknowledged for their toxicity within the training dataset, possibly being a constituent thereof. Furthermore, the 100% prediction accuracy in this instance signifies the ProTox-II model’s proficiency in precisely forecasting the toxicity class and LD50 value for aconitine. The predictions made by the model align seamlessly with the real-world data observed values [41], indicating the robustness and performance of this theoretical model. Moreover, from the analysis depicted in Figure 2C emerges a discernible set of patterns, indicating a predisposition towards inactivity, underscored by notably high probabilities, in some area of toxicological profiling. Notably, the predictions strongly suggest inactivity—expressed as a heightened probability—in the domains of organ toxicity (hepatotoxicity) and both Tox21 pathways (nuclear receptor signaling and stress response pathways). Contrastingly, a conspicuous exception to this trend is evident for immunotoxicity (a high probability of activity is predicted). Moreover, the predictions made for some toxicity endpoints (e.g., carcinogenicity, mutagenicity, and cytotoxicity) indicate inactivity. However, the predictions made for the last three toxicity endpoints have moderate to low probabilities.

Origin recognition complex (ORC) is a highly conserved heterohexameric protein encoded by the latheo gene and is involved in DNA biosynthesis during the cell cycle [47]. Recent molecular docking studies showed that aconitine has a good binding activity with isoform five (ORC5) through two stable hydrogen bonds with GLU502 and ALA542, various van der Waals forces with amino residues and alkyl interactions between the ligand and residues ILE689 and LEU723 [48]. Overall, the nature of structure–activity relationship of aconitine is far from being elucidated.

In traditional processing methods, the preparation of herbal formulations is based on empirical detoxification. Hydrolysis is recognized as the pathway of aconitine detoxification. According to the most recent works, indaconitine, benzoylaconitine, pyroaconitine, aconine and 16-epi-pyroaconine could be the hydrolisates of aconitine, whose toxicity decreases in order of the description [49,50,51] and thus explains the reduced effects of herbal drugs.

### 2.2. Antiarrhythmic Activity of Aconitine in Antagonist Treatment Approaches

#### 2.2.1. Mechanism of Action of Aconitine

Aconitine, as featured in the latest release of the IUPHAR/BPS Guide to PHARMACOLOGY (version 2024.2, on 26 June 2024) [52], is documented as an activator (partial agonist) for voltage-gated sodium channels (Nav) across both human and rat species. These channels serve a pivotal role in the generation and propagation of action potentials within excitable tissues, encompassing the heart and nerves. In the context of human physiology, aconitine functions as a partial agonist for the sodium channel protein type 5 subunit alpha (Nav1.5, UniProtKB Q14524). This activity is observed within a concentration range of 3 × 10^−5^ to 1 × 10^−4^ M, under a voltage of −140.0 mV [53,54]. Concurrently, in rats (Rattus norvegicus Berkenhout, 1769), aconitine acts as a partial agonist for the sodium channel protein type 2 subunit alpha (UniProtKB P04775), exhibiting a dissociation constant (K_d_) value of 1.2 µM [55].

#### 2.2.2. Toxic Effects and Mechanisms

**Figure 3:**
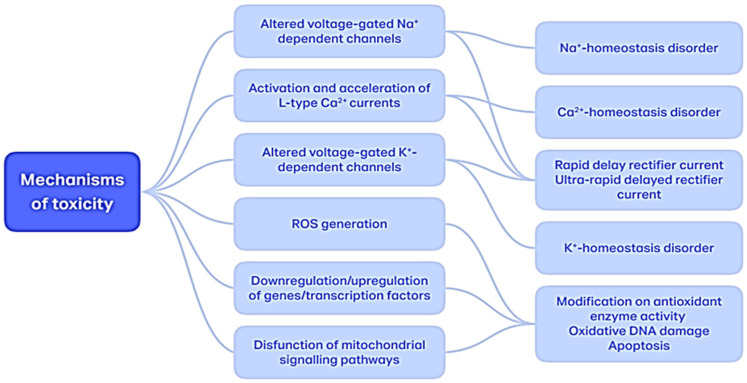
Multifactorial mechanism of aconitine toxicity.

The mechanism by which aconitine exerts its toxic effects is multifactorial (Figure 3), its harmful effect having different degrees of severity: damage to myocardial cell Na^+^ channels, DNA damage, changes in energy metabolism and/or leading to cellular apoptosis [47,56,57].

Na+ overload caused by the aconitine-modified voltage-gated Na^+^-dependent channels induces the shortening of action potential duration (APD), and increased Ca^2+^ provides an arrhythmogenic delayed afterdepolarization (DAD) [58].

Aconitine possesses a high and specific binding affinity for neurotoxin-binding site 2 on the alpha-subunit of the Na^+^ channel protein in cardiac myocytes, muscles and nerves, causing their persistent activation, refraction to a new excitation, and hyperpolarization as a consequence of the massive influx of sodium inside the cells [56]. By the ventromedial nucleus of the hypothalamus activation, aconitine has hypotensive and bradycardic properties [59]. Through its perpetuating action on axon-dependent voltage-gated Na^+^ channels, aconitine blocks neuromuscular transmission by increasing/decreasing the release of acetylcholine levels from the motor nerve [60,61]. The toxic effects of aconitine are also attributed to its cholinolytic properties via vagus nerve excitation [62].

#### 2.2.3. Antagonistic Effects Related to Other Compounds

In 1999, Ameri and Simmet [63] found that neuronal suppression induced by 100 μM aconitine in rat hippocampal slices was reversed by 10 μM lappacontine. The same authors showed that the structurally related alkaloid ajacine has similar activity, but it is a less potent antiepileptiform and sodium channel blocking agent. These results reflect the competitive antagonism of lappaconitine and ajacine on the one side and aconitine on the other side for binding site 2 of the voltage-dependent Na^+^ channel.

Aconitine causes the persistent activation of Na^+^ channels, which become refractory to excitation. A temporary inhibition of aconitine-induced DAD and a decrease in hyperpolarization were induced by acetylcholine in a frog atrium, probably as a result of muscarinic receptor desensitization. It is supposed that tetrodotoxin has a mutual antagonistic effect, inhibiting the Na^+^ inflow when the channel is in an open state [64,65].

Chen et al. [38] found that berberine exhibited a strong antagonistic effect on aconitine-induced arrhythmias in rats treated with Chuan-wu (Crude Radix Aconiti) herbal treatment. Berberine pretreatment inhibited the delayed afterdepolarization induced by the same concentration of aconitine but was inefficient when higher doses of aconitine were intragastrically administered.

Although voltage-gated Na^+^ channels are the main molecular targets of this C19-DA, ex vivo experiments showed aconitine has a strong effect on K^+^-channels as well. Specifically, cardiac human ether-a-go-go related genes (HERGs) encode the pore-forming alpha subunit of voltage-gated K^+^-channels. The K^+^-channels are responsible for the rapid delayed rectifier current (IKr), and Kv1.5 is the channel responsible for the ultra-rapid delayed rectifier current (IKur). Aconitine holds both these channels in an open state, and since the IKr is the major repolarization current of human cardiomyocytes, the blockade of HERGs leads to the further prolongation of the action potential, thus further increasing the arrhythmogenicity of aconitine. Aconitine generates self-exciting microcircuits in the myocardium, leading to arrhythmia and the occurrence of conduction blockages [19,66].

Calcium ions (Ca^2+^) are important secondary messengers, playing a critical role in the translation of cellular signals and in the electrical activity of myocardial cells. The dyshomeostasis of intracellular Ca^2+^ is an important mechanism of arrhythmias caused by aconitine [67]. The arrhythmogenic effect could be the result of increased intracellular Ca^2+^ as a result of the activation and acceleration of L-type Ca^2+^ currents (ICa, L), increasing the expression of NXC and decreasing the expression of SERCA2a and DAD [68].

Calcium-regulating proteins—the sodium/calcium exchanger (NCX1), ryanodine receptor 2 (RyR2), dihydropteridine reductase (DHPR) and cardiac sarcoplasmic reticulum calcium ATPase (SERCA2a) —are responsible for Ca^2+^ homeostasis in heart cells. When applied in a concentration of 120 μmol/L, aconitine affects in vitro myocardial cells by increasing RyR2 expression and decreasing NCX1, DHPR-a1 expression, favoring calcium overload and leading to calcium homeostasis disorder. Aconitine (60 μg) in combination with liquiritin and glycyrrhetinic acid in 1:2:8 and 1:1:1 ratios inhibits the expression of RyR2 and induces the expression of NCX1, DHPR-a1. The above combinations enhanced the contraction of myocardial cells via regulating the expression of L-form voltage-gated calcium channel (DHPR) proteins [69]. Similar results were reported by Zhou et al. [70,71] when male Sprague Dawley rats were treated with aconitine in combination with electroacupuncture. The combined therapies increased the left ventricular systolic pressure, ejection fraction and fractional shortening, up-regulated the expression of SERCA2a and down-regulated the expression of PLB and NCX1 in cardiomyocytes, suggesting a synergistic and attenuating effect of electroacupuncture on aconitine activity.

Zhao et al. [72] revealed that in rat arrhythmia induced via continuous aconitine infusion, arctigenin led to an antiarrhythmic effect by inhibiting the APD, sodium currents (INa) and ICa, L, and enhanced the transient outward potassium current (Ito). A reduction in aconitine toxicity by tetrodotoxin (produced by the pufferfish Fugu) in a dose-dependent manner was also observed by Ohno et al. [64]. By the in vivo co-administration of aconitine and tetrodotoxin to the Institute of Cancer Research (ICR) mice, the toxic signs were markedly reduced, and the mortality was lower.

#### 2.2.4. Therapeutics and Detoxification Strategies

In modern medicine, there is no effective cure for aconitine poisoning. However, supporting treatment for detoxification by charcoal hemoperfusion, amiodarone, magnesium, flecainide, lidocaine or mexiletine [73,74,75,76,77] can be successfully applied, while in traditional medicine, detoxification involves the processing of plant material by soaking, boiling, roasting and steaming.

Acontine and emodin were cytotoxic to interstitial cells of Cajal isolated from the colon of Kunming (KM) mice. Aconitine induced membrane integrity impairment in a time-dependent manner, while emodin was toxic immediately or in a short time after treatment. In the co-exposure experiment of aconitine/emodin in a 2:1 ratio, membrane lipid peroxidation was significantly inhibited and the sodium–potassium adenosine triphosphatase (NA^+^-K^+^-ATPase) activity was not perturbed, revealing an antagonistic effects of emodin that reduced the toxicity of aconitine [78].

The simultaneous administration of 200 μg/kg aconitine with 20 mg/kg peoniflorin in Sprague Dawley (SD) rats reduced the peak plasma concentration and the time-to-peak concentration of aconitine. Also, the administration of 120 and 240 mg/kg bodies of paeoniflorin simultaneously with 1.8 mg/kg aconitine in ICR mice increased the LD50 of aconitine to 2.30 mg/kg and 2.15 mg/kg, and the death rate of mice was reduced in a dose-dependent manner from 50% to 15% [79]. Sweroside is an active compound of Veratrilla baillonii Franch from the Gentianaceae family used as an ethnodrug in the southwest of China. A recent study showed that 50 mg/kg sweroside alleviated the toxicity of aconitine on H9c2 cell lines. Cells treated simultaneously with aconitine and sweroside showed markedly lowerlactate dehydrogenase (LDH) activity compared to cells treated with aconitine alone, suggesting the potential to reduce aconitine toxicity and promote cell survival. The preincubation of H9c2 cells with sweroside in a range of 2–20 μM induced a reduction in the intracellular production of reactive oxygen species (ROS) and the intensification of superoxide dismutase (SOD) activity, as well as a reduction in cell apoptosis, opposite effects compared to the individual treatment with 10 μM aconitine. Sweroside (2–10 μM) markedly inhibited the elevation of the intracellular Ca^2+^ induced by aconitine. It has been noticed that sweroside could down-regulate the L-type voltage-dependent Ca^2+^ channels (LVDCCs), stimulate the activity of SERCA2a and reverse almost completely the increased mRNA expression level of DHPR and RyR2 induced by aconitine [80].

Validation through additional experiments of the above combination therapies could be the beginning of developing novel strategies that have the potential to protect against aconitine-induced myocardial injuries.

### 2.3. Interaction of Aconitine with Antioxidant Enzyme Systems—Synergistic Effects

#### 2.3.1. Overview of Aconitine’s Role in Oxidative Stress

The excessive production of ROS and reactive nitrogen species (RNS) through the oxidative mitochondrial mechanism in the process of cellular respiration and hypoxic conditions, respectively, can lead to an imbalance of cellular homeostasis, the oxidation of proteins, lipids and nucleic acids, and, consequently, an alteration of the signal transduction, gene expression and activation of receptors. Oxidative stress caused by ROS accumulation and ineffective antioxidant systems has been often reported to be involved in the pathogenesis of metabolic disorders such as type 2 diabetes mellitus, obesity, hypertension and cardiovascular diseases [81].

#### 2.3.2. Antioxidant Activity of Aconitine

A limited number of studies reported a potent secondary antioxidant activity of aconitine. For example, Yin et al. [82] claimed that aconitine-type C19-DAs did not exhibit anti-radical activity but were appreciated as a potent secondary antioxidants for their strong binding effects to metal ions (Fe^2+^-chelating activity). In another study, aconitine, mesaconitine and hypaconitine from Fuzi extract increased the efficiency of SOD enzymatic systems after the substrate was deactivated in lipid peroxidation reactions, a condition that can contribute to a reduction in serum malondialdehyde (MDA) levels [83]. Park et al. [84] recorded a decrease in NO levels in LPS- or IFN-γ-stimulated tumor necrosis factor alfa (TNF-α) and nitric oxide in Raw 264.7 macrophage after treatment with 1–100 mg/kg aconitine, along with a slight increase in cytotoxicity (AC 1000 ≤ μg/mL).

#### 2.3.3. Aconitine-Induced Oxidative Stress

Conversely, many studies have shown that aconitine stimulates the production of ROS, increasing the content of MDA and reducing SOD activity in cardiomyocytes, significantly increased the content of 8-hydroxy-2′-deoxyguanosine (8-OHdG) and reduced the γ-glutamyl-cysteinyl-glycine (GSH) levels, thus leading to an intracellular imbalance between the antioxidant systems and ROS and to apoptosis [57,67,79,85,86]. An increased release of cytochrome c from mitochondria and an intensification of apoptosis was reported after treating hepatic carcinoma cells (HepG2 and Hub7) and H9c2 cardiac cells with aconitine [67,87].

#### 2.3.4. Synergistic Effects of Aconitine with Other Compounds

The combined treatment of quercetin and aconitine revealed a synergistic inhibitory effect on HeLa cell viability. When compared with each treatment alone, the combination of quercetin and aconitine in a varying concentration of 0–61.75 μg/mL and 1:1 concentration ratio induces a 0.78-fold and 0.61-fold decrease in IC50, and the combination index ranges from 0.1 to 0.6. It has been reported that the combined treatment of quercetin and aconitine synergistically decreased cellular growth and the expression of the multidrug resistance protein 1 (MDR1) mRNA level, increased alF2a and ATF4 mRNA expression, causing an accumulation of unfolded proteins in the endoplasmic reticulum through the protein kinase R-like endoplasmic reticulum kinase (PERK) pathway, induced ROS generation and mitochondrial membrane and DNA damages, and stimulated apoptosis in HeLA cells [88].

Recent studies have shown that aconitine stimulates the accumulation of ROS, oxidative DNA damage, autophagy and apoptosis. The oxidative stress induced by aconitine was confirmed by the in vitro and/or in vivo promotion of lipid peroxidation, oxidation of DNA nucleosides, down-regulation of oxidative stress-related genes, phosphorylation of adenosine 5′-monophosphate-activated protein kinase (AMPK) and autophagy-initiating regulator ULK1, and up-regulation of the expression of pro-apoptotic genes and targeting transcription factors such as nuclear factor-kappa B (NF-kB) [57,89,90].

### 2.4. Antiinflamatory and Antiproliferative Effect of Aconitine in Combined Treatments

Initially well-known for its role in defending the body against pathogens, in repairing tissues and regulating tissue homeostasis, the relationship between the inflammation mechanism, immune system and cancer promotion it is currently a certainty proven by numerous studies [91,92,93]. Furthermore, He et al. [94] showed that the combination of active ingredients of liquorice (liquiritin, the liquiritigenin group, glycyrrhizic acid, glycyrrhetinic acid) with aconitine induces synergic effects on induced hind paw edema in Sprague Dawley rats. The increased anti-inflammatory effect of aconitine combined with active ingredients of liquorice was explained by the in silico results, based on which the authors reported that liquorice inhibited the function of the key transporter P-glycoprotein, while aconitine acted as a substrate of proteins.

Mouse macrophage cell line Raw 264.7, mimicking the inflammatory response characteristic of rheumatoid arthritis, was treated with a combination of aconitine and methotrexate at a 3:1 ratio. Synergistic and additive decreasing effects on levels of TNF-α and inflammatory cytokines (IL-1α, IL-6 and RANTES) in a dose-dependent manner and a decrease in extracellular signal-regulated kinase, c-Jun N-terminal kinase and the pp38 protein involved in the MAPK-dependent pathways were noticed after the combined treatment [84].

Aconitine has proved effective in vitro anti-tumor activity by inducing the apoptosis of tumor cells and DNA damage (Table 2). However, at present, the mechanisms are unclear and require additional studies. Combined treatment with osthole, psoralen and aconitine in concentrations of 6.44, 8.89 and 9.44 μg/mL for 24 h significantly inhibited the invasion of human breast cancer MDA-MB-231BO cell lines. It has been noticed that transforming growth factor beta 1 (TGF-β1), mothers against decapentaplegic homolog (Smad)—Smad2, Smad3, Smad4 and Smad7—Nuclear factor kappa B (NF-κB) and receptor activator of nuclear factor-κB (RANK) mRNA expressions were significantly inhibited by this combination [95].

In combination with crude monkshood polysaccharide, aconitine enhanced the ability of the immunocytes to kill the in vitro hepatocellular carcinoma cells, elevated CD4^+^ T and CD8^+^ T cells and macrophage in the spleen, decreased serum IL-6 levels and increased serum interferon gamma (IFN-γ) and TNF-α levels in mice. Therefore, the crude monkshood polysaccharide and aconitine had an additive effect on the anti-tumor immune response [97].

The caspase inhibitor benzyloxycarbonyl-Val-AlaAsp-fluoromethylketone (pan-caspase inhibitor Z-VAD-FMK) together with 50 μg/mL of aconitine decreased the apoptotic rate in HepG2 cells to a level similar to that of control cells, while the inhibitor alone had little effect on the cell apoptotic rate and aconitine alone significantly increased the apoptotic rate [87].

### 2.5. Reduction in Aconitine Toxicity by Entrapment in Designed Nanoparticles

Known for its hydrolysis-induced instability and low solubility in water or accepted organic solvents, treatment with aconitine may have low efficacy or lead to adverse effects [51,67]. In order to overcome these obstacles, regulate drug release, modulate biodistribution and improve bioavailability, and expand or reduce the range of its effects, aconitine has been encapsulated in various nanoparticles (NPs). The pharmacokinetic behavior of aconitine in a herbal formulation could be improved by other ingredients via a synergistic mechanism or by entrapment in engineered NPs. The co-treatment of aconitine with both liquiritin and 6-gingerol on Caco-2 cells enhances the absorption of aconitine and its blood concentration by inhibiting the activity of P-glycoprotein (P-gp) [98]. For example, aconitine-loaded poly(d,l-lactide-coglycolide) nanoparticles could lead to an improvement in the stability of aconitine and in vitro sustained release from nanoparticles [99].

The encapsulation of aconitine in self-assembled liquorice protein (a boiling-stable 31 kDa protein purified from licorice) nanoparticles (238.2 ± 1.2 nm) determined a significant attenuation of toxicity, compared to aconite decoction, which induced the death of 100% ICR mouse after intraperitoneal injection [100].

Aconitine loaded in microemulsion in the form of spheroidal droplets with a size distribution of less than 100 nm was administered using microneedles. The use of microemulsion as a nanocarrier reduced the cytotoxicity of aconitine in HaCaT cells and CCC-ESF-1 cells, improved delivery due to the sustained release of this fat-soluble alkaloid, and improved transdermal absorption by topical administration [101].

As a matter of fact, nanotechnology is well known as one of the ways that can reduce toxicity and lead to significant progress in various therapies [24,26] (and may harmoniously complete the picture of clinical uses of Aconitum preparations).

## 3. Conclusions and Perspectives

This review provides a comprehensive overview of the multifaceted impacts of aconitine, an alkaloid derived from the Aconitum species, particularly emphasizing its synergistic, additive, and antagonistic interactions in combination therapies, as gathered from 30 specific studies.

The evidence indicates that aconitine, when utilized in conjunction with various bioactive compounds, demonstrates promising synergistic effects that can enhance its therapeutic efficacy while simultaneously mitigating its toxicological risks. Notably, the entrapment of aconitine in engineered nanoparticles provides a novel avenue for improving its bioavailability and reducing its adverse effects, paving the way for safer medicinal applications. This underscores the importance of interdisciplinary approaches, integrating molecular biology, pharmacology, and nanotechnology, to optimize the use of aconitine and similar compounds in healthcare.

Moreover, this review reveals the need for further research to elucidate the complex mechanisms underlying the various interactions of aconitine within biological systems. Specifically, targeted studies focusing on the dose–response relationships, long-term effects of combination therapies, and the intricate pathways of aconitine’s action can help to expand our understanding of the dichotomy in the pharmacological profile of aconitine and enable the development of tailored therapies that harness its beneficial properties while minimizing its associated risks.

As practitioners and researchers move toward more integrative approaches in treating multifactorial diseases, the therapeutic potential of aconitine warrants further exploration. Building upon the existing knowledge base will require a collaboration across multiple disciplines—including clinical pharmacology, toxicology, and herbal medicine practices—to formulate evidence-based guidelines that maximize patient safety and treatment outcomes. This exploration may not only contribute to the development of effective therapeutic strategies involving aconitine but may also shed light on the broader implications of utilizing plant-derived biochemical compounds in modern medicine.

The evaluation and validation of the synergistic, additive and antagonistic effects of drug combinations using adequate reference models and appropriate approaches (in vitro, in silico, in vivo, and clinical trials), as well as balancing aconitine’s efficacy and toxicity, remain pivotal considerations. Future investigations should aim to refine treatment modalities, improve patient safety, and enhance the utility of aconitine in clinical settings by navigating its complex pharmacological landscape through innovative and safe records.

## 4. Methodology

The present study systematically investigated the synergistic and additive effects of aconitine in conjunction with other bioactive compounds, drugs and/or drug delivery systems. A comprehensive search strategy was used across the following databases: Web of Science, Scopus, Science Direct, Dove Medical Press, PubMed and Google Scholar. Additionally, regarding the PubMed database, we used the controlled vocabulary thesaurus, the Medical Subject Headings—MeSH terms. The use of MeSH terms ensured that we took a precise and systematic approach to information retrieval and indexing in the context of our review. The relevant information for Romanian folk and homeopathic medicine was extracted from books available in the library of the Department of Natural Sciences, the National University of Science and Technology POLITEHNCA Bucharest.

Following the curation process, a total of 99 relevant papers from the last thirty years used in this review were selected based on a controlled vocabulary that included strictly the keywords aconitine, synergistic, additive, antagonism, combination therapy, oxidative damage, DNA damage, nanoparticles—used individually or grouped in pairs. Emphasis was placed on selecting studies that explored these terms either individually or in paired combinations. Meta-analyses and (systematic) reviews were not considered for this work.

## Figures and Tables

**Table 2:** Aconitine induced in vitro DNA damage and apoptosis.

In Vitro/In Vivo Studies	Effect	Underlying Mechanism	Approach for Assessing	Concentration of Aconitine	Exposure Time	Reference
H9c2 cardiac cells	Oxidative DNA damage (increased levels of ROS and 8-OhdG) Apoptosis	MAPK activation pathway and ULK1 phosphorylation	Enzyme-linked immunosorbent assay and Western blot analysis Flow cytometry	50, 100 or 200 μM	24 h	Wang et al. [57]
Human OVCA A2780 cell line	Apoptosis and DNA damage	Mitochondrial pathways: changes in mitochondrial membrane potential and permeability via up-regulation of expression levels of p53, cytochrome C, cleaved caspase-3/9, down-regulation of expression levels of Bax/Bcl-2, Bcl-xl, Apaf-1, PARP, MMP2, MMp9 and p-ATM	MTT assay Comet assay	10, 50, 100, 200, 400, 800 and 1000 µg/mL 100, 200 and 400 µg/mL	24 h	Wang et al. [47]
H9c2 cardiac cells	Apoptosis	Mitochondria-mediated signaling pathways: down-regulation of Bcl-2, up-regulation of Cytochrome c, caspase-3, decreased level of PGc-1α	Fluorescence microscopy Flow cytometry	100 and 200 µM	24 h	Gao et al. [67]
HepG2 (human hepatocellular carcinoma cells)	Apoptosis	Depolarization of mitochondrial membrane via 2–3-fold increased production of intracellular ROS and release of cytochrome C from mitochondria in cytosol	MTT assay Annexin V-fluorescein isothiocyanate (FITC)/propidium iodide (PI) double-staining assay	6.25, 12.5, 25, 50, 100 µg/mL	24, 48 and 72 h	Qi et al. [87]
Huh7 (human hepatocellular carcinoma cells) in mice induced 5 mm diameter tumors	In vivo and in vitro inhibition of tumor cell proliferation	NA	Tumor tissue sections stained with hematoxylin and eosin Evaluation of tumor size	2.4 mg/kg	21 days after aconitine injection into the tumors	Qi et al. [87]
Rat myocardial cells H9c2	DNA damage Apoptosis	Intercalation in DNA double helix, coupled with electrostatic interaction	MTT assay Flow cytometry	12.0 × 10^−8^ M (UV-Vis analysis), 4.0~16.0 × 10^−5^ M (Fluorescence and melting studies)	24 h	Liu et al. [49]
B16, mouse melanoma cells and B16’s metastatic derivative cells B16F1 and B16F10	Inhibition of cell growth rates and apoptosis	PI3K/AKT and MAPK/ERK1/2 signaling pathway inhibition: AKT and ERK1/2 inactivation, protein levels of PCNA suppression, Caspase-3 and Caspase-8 activation Reduced tumor weights	In vitro chemosensitivity assay Annexin-V apoptotic assay Western blotting assay In vivo study	In vitro: 6.25 and 12.5 µg/mL In vivo: 0.06 mg/kg/d 0.12 mg/kg/d	48 h in vitro Five weeks in vivo	Du et al. [96]

## Data Availability

The original contributions presented in this study are included in the article. Further inquiries can be directed to the corresponding author.

## Abbreviations

8-OHdG	8-hydroxy-2′-deoxyguanosine
AMPK	adenosine 5′-monophosphate-activated protein kinase
APD	action potential duration
C_19_-DA	C_19_-diterpenoid alkaloid
DAD	delayed after depolarization
DAs	diterpene alkaloids
DHPR	dihydropteridine reductase
DNA	deoxyribonucleic acid
GSH	γ-glutamyl-cysteinyl-glycine
HERG	human ether-a-go-go-related genes
ICa, L	L-type calcium current
IFN-γ	Gamma interferon
Ikr	rapid delayed rectifier current
IKur	ultra-rapid delayed rectifier current
Ina	sodium currents
K_d_	dissociation constant
KM	Kunming
LD	lethal dose
LDH	lactate dehydrogenase
LPS	lipopolysaccharide
LVDCCs	L-type voltage-dependent Ca^2+^ channels
MDA	malondialdehyde
MDR1	multidrug resistance protein 1
mRNA	messenger ribonucleic acid
NA^+^-K^+^-ATPase	sodium–potassium adenosine triphosphatase
NCX	Na^+^-Ca^2+^ exchanger
NF-kB	nuclear factor-kappa B
NPs	nanoparticles
ORC	origin recognition complex
PERK	protein kinase R-like endoplasmic reticulum kinase
PLB	phospholamban
RANK	receptor activator of nuclear factor-κB
RNS	reactive nitrogen species
ROS	reactive oxygen species
RyR2	ryanodine receptor 2
SD	Sprague Dawley
SERCA2a	sarcoplasmic reticulum Ca^2+^ATPase 2a
Smad	mothers against decapentaplegic homolog
SOD	superoxide dismutase
TGF-β1	transforming growth factor beta 1
TNF-α	tumor necrosis factor-alpha
